# Effects of Preoperative Postauricular Glucocorticoid Injection on Electrode Impedance in Cochlear Implantation

**DOI:** 10.3390/healthcare14070922

**Published:** 2026-04-01

**Authors:** Linsui Wu, Ting Zhang, Hongyi Peng, Yufeng He, Shixun Zhong

**Affiliations:** 1 Department of Otolaryngology, The First Affiliated Hospital of Chongqing Medical University, Chongqing 400016, China; wulinsui589@163.com (L.W.); 2023110252@stu.cqmu.edu.cn (T.Z.); 2024140170@stu.cqmu.edu.cn (Y.H.); 2 Department of Otolaryngology of Jiangbei Campus, The First Affiliated Hospital of Army Medical University, Chongqing 400020, China; 2021120244@stu.cqmu.edu.cn

**Keywords:** electrode impedance, cochlear implantation, glucocorticoid therapy, postauricular injection

## Abstract

**Objectives**: We aimed to investigate the short-term effects of preoperative postauricular glucocorticoid (GC) injection on electrode impedance in cochlear implant (CI) recipients. **Methods**: A total of 69 participants were enrolled: 44 children (<18 years) and 25 adults (18–85 years). Using a pre-specified non-randomized alternating assignment strategy, they were respectively assigned to either the treatment group (preoperative postauricular methylprednisolone injection and intraoperative intratympanic betamethasone) or the control group (intraoperative intratympanic betamethasone alone). Electrode impedance was measured intraoperatively and at 1, 3, and 6 months postoperatively. Owing to the use of different implant systems in pediatric and adult patients, the two cohorts were analyzed separately. Longitudinal impedance data across cochlear turns (apex, middle, base) were analyzed using linear mixed-effects models adjusted for baseline values. This study was registered on Chictr.org.cn (ChiCTR2400081024). **Results**: In the pediatric cohort, a significant interaction between group and time was observed (F = 8.34, *p* < 0.001); however, post hoc analyses did not demonstrate statistically significant differences between groups at individual postoperative time points (all *p* > 0.05). In the adult cohort, a significant interaction between group and turn was identified (F = 3.07, *p* = 0.049); post hoc analysis demonstrated statistically significant differences in impedance in the middle turn between groups (intervention effect = 1.355 kΩ; 95% CI, 0.115 to 2.596; *p* = 0.033). **Conclusions**: Preoperative postauricular GC administration, when combined with intraoperative intratympanic steroid therapy, may be associated with differences in postoperative electrode impedance dynamics and the electrode–tissue interface.

## 1. Introduction

Cochlear implantation (CI) has revolutionized the rehabilitation of patients with severe to profound hearing loss, serving as the first successful implantable brain nerve stimulator [1]. Despite its success, preserving hearing after implantation remains a challenge, as it is compromised by both acute and delayed pathological processes. Acute hearing loss occurs primarily in the early stage due to mechanical trauma during electrode insertion [2],while delayed hearing loss involves a more complex etiology, including a foreign body rejection response, fibrous tissue formation, and eventual ossification in the cochlea [3,4,5,6]. These factors collectively contribute to the long-term performance variability of CIs, which has become a focal point of recent clinical research [7].

Glucocorticoid (GC), renowned for its potent anti-inflammatory and immunosuppressive effects, has emerged as a promising pharmacological intervention to mitigate these adverse effects. The presence of GC receptors in the cochlea substantiates their efficacy in reducing edema, suppressing inflammation, and enhancing local microcirculation [8,9]. Consequently, various routes of GC administration, including systemic (intravenous/oral), intratympanic, intracochlear, and electrode-loaded delivery, have been explored to alleviate insertion trauma and inhibit the chronic inflammatory cascade that leads to fibrosis and ossification [10,11,12,13]. Among commonly used agents, methylprednisolone is widely applied in inner ear disorders because of its potent anti-inflammatory effects and favorable safety profile, whereas betamethasone is frequently used for intratympanic administration due to its strong anti-inflammatory activity and prolonged local effect [14,15].

Postauricular injection could potentially serve as an effective administration route for the inner ear, featuring the merits of minimal invasiveness, convenience, and a high local drug concentration in the cochlea [16,17]. This approach has proven effective in treating sudden sensorineural hearing loss and Bell’s Palsy [18,19]. Nevertheless, to our knowledge, the effect of preoperative postauricular GC administration on postoperative electrode impedance in cochlear implantation has not been previously investigated, particularly with respect to its potential role as a perioperative strategy for modulating postoperative electrode impedance.

We hypothesize that adding a preoperative postauricular GC injection to routine intraoperative intratympanic steroid administration may help attenuate postoperative increases in electrode impedance compared with intratympanic therapy alone.

Therefore, this prospective study aims to evaluate the impact of this combined GC regimen on electrode impedance dynamics by measuring short-term electrode impedance changes—a reliable indicator of the electrode–nerve interface and intracochlear status in both pediatric and adult CI recipients [20,21].

## 2. Materials and Methods

### 2.1. Participants and Group Allocation

This prospective, non-randomized controlled cohort study was conducted at the Department of Otolaryngology, The First Affiliated Hospital of Chongqing Medical University, from 2021 to 2023. Participants were eligible for inclusion if they met the indications for cochlear implantation, as outlined in the 2013 Chinese Clinical Guidelines for Cochlear Implantation [22], agreed to participate in the study and committed to complying with the follow-up schedule. Exclusion criteria included contraindications to GC therapy. Patients with well-controlled hypertension or diabetes were not excluded. Participants who were lost to follow-up were excluded from the final analysis.

Eligible subjects were consecutively enrolled during the study period and initially stratified into two cohorts according to age: a pediatric cohort (less than 18 years) and an adult cohort (18 years or older).

Within each age cohort, group allocation followed a pre-specified systematic alternation based on surgical date. Patients undergoing surgery on the first scheduled surgical day were assigned to the control group, those on the subsequent surgical day to the treatment group, and this alternating sequence was maintained throughout the study period. The allocation rule was established prior to study initiation and was not influenced by patient characteristics or disease severity.

### 2.2. Intervention and Surgical Procedures

#### 2.2.1. Glucocorticoid Treatment Protocol

The core intervention entailed distinct perioperative GC regimens.

Treatment Group: Administered a combined GC regimen, which included a preoperative postauricular injection of methylprednisolone sodium succinate (40 mg/mL) 24 h before surgery—0.5 mL for children and 1.0 mL for adults—along with an intraoperative intratympanic application of 1.0 mL betamethasone (5 mg/mL) at the round window during cochlear implant surgery.

Control Group: Received the intraoperative intratympanic application of 1.0 mL betamethasone (5 mg/mL) alone following the same procedure.

#### 2.2.2. Surgical Technique

All cochlear implantation procedures were performed by a single senior surgeon to minimize technical variability. Under general anesthesia, a standard post-auricular C-shaped incision was created. A mastoidectomy was conducted, followed by a posterior tympanotomy to gain access the facial recess and visualize the round window membrane.

In all cases, a cochleostomy was created anteroinferior to the round window membrane, approximately 0.5–1.0 mm from the margin of the round window niche, to access the scala tympani. Betamethasone was administered according to the protocol. The implant receiver–stimulator was fixed in a subperiosteal pocket, and the electrode array was slowly advanced into the cochlea using a soft-surgery technique to minimize insertion trauma. Full electrode insertion was achieved in all cases as confirmed intraoperatively and by postoperative radiographic imaging.

Pediatric patients were implanted with the MED-EL SONATA system using FLEX26 (MED-EL, Innsbruck, Austria) electrodes (26 mm). Adult patients received the Cochlear™ Nucleus CI512 system (Cochlear Limited, Sydney, Australia) with a 25-mm electrode array.

No major modifications in surgical technique occurred during the study period.

#### 2.2.3. Antibiotic Prophylaxis

All patients were administered perioperative intravenous cefuroxime sodium for the purpose of infection prophylaxis. The pediatric dosage was set at 60 mg/kg/day, whereas adults received 1.5 g twice daily, and this regimen was maintained for two days. After discharge, an oral course of cefuroxime (250 mg daily) was prescribed for a duration of seven days. This regimen reflects the routine institutional protocol for infection prophylaxis in cochlear implantation.

### 2.3. Data Collection

Demographic and clinical data were collected from all subjects, including age, sex, duration of deafness, history of hearing aid use, etiology of hearing loss and chronic illnesses. Preoperative evaluations included pure-tone audiometry, computed tomography of the temporal bone and magnetic resonance imaging of the internal auditory canal. A postoperative cranial X-ray (Mayer’s view) was acquired to confirm electrode placement. The following parameters were recorded postoperatively: laterality (side of implantation), electrode model, the assigned GC regimen, electrode impedance values, and any surgical or device-related complications.

The primary outcome measure was electrode impedance. Impedance measurements were obtained at four time points: intraoperatively, and at 1 month (initial activation), 3 months, and 6 months after surgery.

Impedance values were recorded for each individual electrode contact during routine telemetry measurements. To evaluate regional impedance patterns along the cochlea, electrodes were grouped into basal, middle, and apical turns according to their relative position along the electrode array. For the MED-EL FLEX26 (12 contacts), contacts 1–3, 4–7, and 8–12 corresponded to the apical, middle, and basal turns, respectively; for the Cochlear™ CI512 (22 contacts), contacts 1–7, 8–14, and 15–22 corresponded to the apical, middle, and basal turns, respectively. Mean impedance for each turn was calculated as the arithmetic average of all contacts assigned to that turn.

Because different cochlear implant systems were used in pediatric and adult cohorts, impedance measurements were obtained using manufacturer-specific clinical software: MAESTRO 8.0 (MED-EL, Innsbruck, Austria) for pediatric patients and Nucleus 1.4 (Cochlear Limited, Sydney, Australia) for adult patients. All impedance measurements were performed according to the manufacturers’ standard telemetry protocols.

### 2.4. Blinding and Bias Control

To minimize potential bias under the constraints of non-random allocation, procedural roles were separated. The surgical procedure was performed by a dedicated surgeon who was not involved in impedance measurement or data analysis. The informed consent process, perioperative postauricular injection, and clinical data recording were conducted by a second group of investigators. Impedance measurements were obtained by a licensed audiologist who was blinded to group allocation. Data analysis was performed using coded datasets without group identifiers.

### 2.5. Statistical Analysis

Statistical analyses were performed using SAS version 9.4 (SAS Institute, Cary, NC, USA). Continuous variables are presented as mean ± standard deviation, and categorical variables as number (*n*, %).

To evaluate potential selection bias associated with the non-randomized allocation, baseline characteristics between the treatment and control groups were compared.

Longitudinal electrode impedance measurements across postoperative time points and cochlear turns were analyzed using linear mixed-effects models. Subject ID was included as a random intercept to account for within-subject correlations. Fixed effects included baseline impedance, group (control vs. treatment), cochlear turn (base, middle, apex), and postoperative time point (1, 3, and 6 months), along with their two-way interaction terms.

Degrees of freedom were estimated using the Kenward–Roger method. Model assumptions were assessed using residual diagnostics. The intervention effect was defined as the model-estimated adjusted difference between the control and treatment groups (control-treatment; positive values indicate lower impedance in the treatment group). A two-sided *p* value < 0.05 was considered statistically significant.

Because different cochlear implant systems and impedance measurement platforms were used in pediatric and adult cohorts, all statistical analyses were performed separately within each cohort to minimize device-related confounding.

## 3. Results

### 3.1. Patient Demographic and Baseline Characteristics

Initially, 80 patients were assessed for eligibility for cochlear implantation. Five patients were excluded prior to enrollment (three met exclusion criteria with contraindications to GC therapy and two declined participation), leaving 75 participants who were consecutively enrolled in the study.

Within each cohort, patients were allocated to the control or treatment groups according to the predefined alternating allocation rule. In the pediatric cohort, 24 patients were assigned to the control group and 24 to the treatment group. In the adult cohort, 15 patients were assigned to the control group and 12 to the treatment group.

During follow-up, 4 pediatric participants and 2 adult participants were lost to follow-up. These participants resided outside the region and subsequently sought follow-up care in local hospitals, making it impossible to complete protocol visits at our center. No withdrawals were related to treatment-related adverse events. Consequently, these participants were not included in the final analysis.

**Figure 1:**
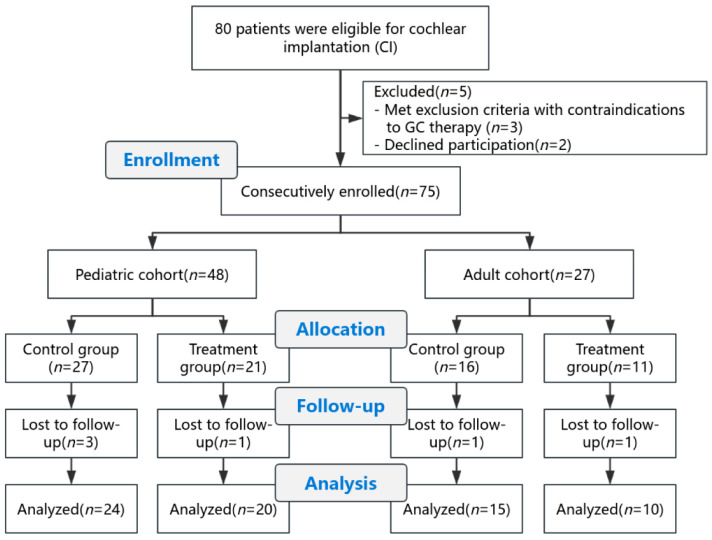
Study cohort flow diagram detailing screening, eligibility, enrollment, and follow-up.

Consequently, 69 participants were included in the final analysis, comprising 44 pediatric patients (control: *n* = 24; treatment: *n* = 20) and 25 adult patients (control: *n* = 15; treatment: *n* = 10) (Figure 1). No surgical complications, such as electrode array mis-insertion, perilymph leakage, or facial nerve stimulation, were observed in any participant. Furthermore, no instances of short circuits, open circuits, or abnormally high electrode impedances were encountered throughout the study period.

Detailed demographic and clinical characteristics are presented in Table 1 (Children) and Table 2 (Adults). In both cohorts, the treatment and control groups were well-matched at baseline, showing no statistically significant differences in age, sex distribution, laterality of implantation, duration of deafness, prevalence of residual hearing, history of hearing aid use, or etiology of hearing loss.

### 3.2. Temporal Trends in Electrode Impedance

**Figure 2:**
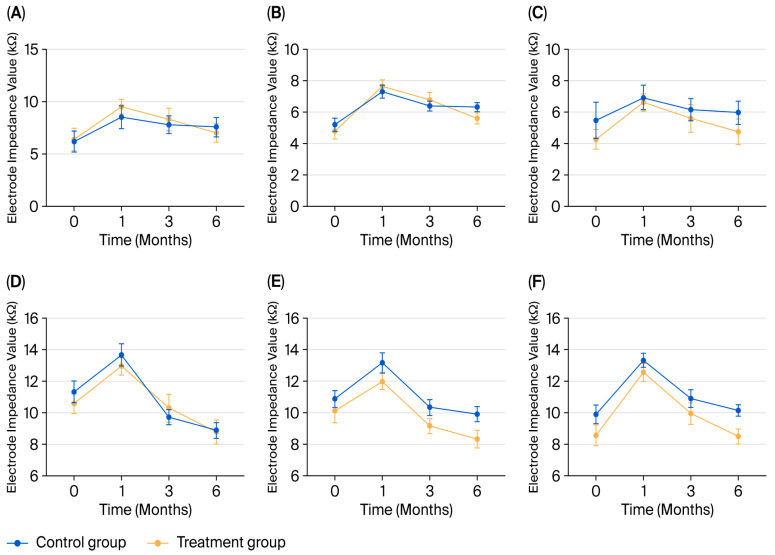
Electrode impedances across cochlear turns (from apex to base) over time in children (**A**–**C**) and adults (**D**–**F**).

Across both pediatric and adult cohorts, electrode impedance in the different turns followed a similar temporal trajectory (Figure 2; Table 3 and Table 4). Impedance values were lowest immediately after implantation, peaked at the initial activation session (1 month postoperatively), and subsequently declined gradually over time, indicating a dynamic process around the electrode array.

### 3.3. Pediatric Cohort: A Mixed Model Analysis of Impedance Profiles Across Cochlear Turns

A linear mixed-effects model was applied to evaluate the effects of perioperative GC administration on postoperative impedance values in the pediatric cohort (Table 5).

A significant effect of turn (F = 105.36, *p* < 0.001) and time (F = 54.27, *p* < 0.001) was observed, whereas no significant main effect of group was observed (*p* = 0.650). The interaction between group and time was statistically significant (F = 8.34, *p* < 0.001), indicating that the influence of GC administration varied across postoperative time points. This finding suggests that impedance changes over time differed between groups

Post hoc analyses showed that no statistically significant difference was observed at 1 month and 3 months (*p* > 0.05; Table 6). At 6 months, a positive intervention effect was observed (estimate = 0.50, 95% CI: −0.39 to 1.40, *p* = 0.264), suggesting numerically lower impedance in the treatment group; however, this difference did not reach statistical significance.

### 3.4. Adult Cohort: A Mixed Model Analysis of Impedance Profiles Across Cochlear Turns

A linear mixed-effects model was similarly applied in the adult cohort to evaluate postoperative impedance dynamics (Table 7).

A significant time effect was observed (F = 103.28, *p* < 0.001), consistent with the typical postoperative evolution of impedance. The interaction between group and turn reached statistical significance (F = 3.07, *p* = 0.049), indicating that the effect of GC differed across cochlear regions.

Post hoc analysis revealed a statistically significant positive intervention effect in the middle turn (estimate = 1.355 kΩ; 95% CI, 0.115 to 2.596; *p* = 0.033; Table 8), indicating lower impedance in the treatment group. No significant differences were observed in the apical or basal turns, but a numerically lower impedance was noted in the basal turn of the treatment group compared to controls.

These findings suggest a region-specific association between GC administration and reduced impedance values, particularly in the middle turn.

### 3.5. Sensitivity Analyses

To evaluate the robustness of the primary findings, several sensitivity analyses were conducted using alternative model specifications (Appendix A Table A1 and Table A2).

Across these alternative analytical approaches, the group-by-time interaction in the pediatric cohort remained consistently significant, supporting the robustness of the observed time-dependent effect.

In the adult cohort, the group-by-turn interaction remained significant or marginally significant in most model specifications, further supporting the stability of the region-specific effect.

Together, these sensitivity analyses indicate that the main findings were not dependent on a single modeling approach, thereby strengthening the reliability of the results.

## 4. Discussion

The present study investigated whether the addition of a preoperative postauricular GC injection to the standard intraoperative intratympanic steroid administration could influence postoperative electrode impedance following cochlear implantation. Using longitudinal mixed-effects modeling, differences in impedance dynamics between groups were observed. These findings indicate that perioperative GC administration may be associated with variations in the electrode–tissue interface.

Electrode impedance represents a key electrophysiological parameter routinely monitored in cochlear implant recipients. In addition to confirming device integrity, impedance measurements provide indirect information about the biological environment surrounding the electrode array [20,21]. Increased impedance values are generally associated with inflammatory reactions, fibrotic tissue proliferation, and changes in ionic composition at the electrode–tissue interface [20]. Histopathological investigations have demonstrated that cochlear implantation may trigger a foreign-body response characterized by inflammatory cell infiltration, fibroblast proliferation, and fibrous tissue formation around the electrode array [3,23]. These biological processes are considered major contributors to postoperative impedance elevation.

The temporal evolution of impedance observed in the present study is consistent with the typical postoperative impedance trajectory reported in previous cochlear implantation studies [24,25]. Impedance values are generally lowest intraoperatively, increase during the early postoperative period around device activation, and subsequently decline during follow-up as the intracochlear environment stabilizes [23]. The early postoperative increase has been attributed to inflammatory responses and the formation of protein layers on electrode contacts, whereas the gradual decrease during later follow-up may reflect stabilization of the electrode–tissue interface and adaptation of the surrounding cochlear microenvironment [2,3,5,10].

GCs have been widely investigated as a potential strategy to reduce intracochlear inflammation associated with cochlear implantation. Experimental studies have demonstrated that steroids can suppress inflammatory cytokine expression, reduce macrophage infiltration, and limit fibrotic tissue formation within+ the cochlea [8,9]. In animal models, local steroid administration has been shown to attenuate insertion-related trauma and preserve cochlear structures following electrode implantation [26]. These anti-inflammatory effects may contribute to a more stable electrode–tissue interface and potentially influence postoperative impedance dynamics.

In clinical practice, steroids may be delivered through several routes, including systemic administration, intratympanic injection, and regional delivery strategies. Intratympanic steroid administration is widely used during cochlear implantation because it allows for relatively direct drug delivery to the inner ear while minimizing systemic exposure [27]. In the present study, intratympanic betamethasone application represented the standard perioperative anti-inflammatory protocol administered to all patients. The intervention investigated in this study was the additional preoperative postauricular injection of methylprednisolone.

Postauricular steroid injection has been proposed as a regional drug delivery approach for inner-ear disorders [17]. Pharmacokinetic studies suggest that drugs administered in the postauricular region may reach the inner ear through a combination of local diffusion pathways and systemic distribution via the blood–labyrinth barrier [16]. Compared with systemic steroid therapy, this regional delivery method may achieve higher inner-ear drug concentrations while reducing systemic adverse effects. Furthermore, current evidence suggests that postauricular steroid administration does not produce significant concentration gradients across the cochlear turns, potentially resulting in a more homogeneous drug distribution—a therapeutic advantage for diffuse inflammatory conditions [16]. However, the exact pharmacokinetic mechanisms and their relevance to clinical outcomes remain to be clarified.

In the present study, impedance modulation appeared more pronounced in the basal and middle turns. This pattern may reflect the anatomical proximity of these regions to the site of electrode insertion, where surgical trauma and subsequent inflammatory responses are expected to be most prominent. Previous histopathological studies have reported that tissue reactions following cochlear implantation are typically greatest near the cochleostomy or round-window region corresponding to the basal turn [3,23]. Consequently, anti-inflammatory interventions may have a relatively greater influence in these regions.

Although the observed impedance differences suggest that perioperative GC administration may influence the electrode–tissue interface, the clinical significance of these findings remains uncertain. Impedance measurements primarily reflect electrophysiological characteristics of the local tissue environment rather than direct indicators of auditory performance. Previous studies have suggested that the relationship between impedance values and cochlear implant performance parameters, such as speech perception outcomes or stimulation thresholds, is complex and not necessarily linear [28]. Therefore, reductions in impedance should be interpreted cautiously and should not be regarded as direct evidence of improved auditory outcomes.

Several limitations of the present study should be acknowledged. First, the overall sample size was relatively modest, particularly in the adult treatment subgroup, which may limit statistical power for detecting small-to-moderate effects. Second, functional auditory outcomes such as speech perception scores, stimulation thresholds, and programming parameters were not prospectively collected, preventing direct evaluation of the relationship between impedance dynamics and auditory performance. Third, pediatric and adult patients were implanted with different cochlear implant systems, which may introduce heterogeneity related to electrode design, surface materials, and impedance measurement algorithms. Although statistical analyses were conducted separately within each cohort to minimize cross-platform confounding, this factor may still influence impedance characteristics. Finally, the study employed a systematic alternation allocation strategy rather than randomization, which may introduce residual selection bias despite the predefined allocation strategy and baseline comparability.

Future studies with larger cohorts, standardized implant systems, and longer follow-up periods are needed to further investigate the impact of perioperative steroid strategies on intracochlear tissue responses. In particular, integrating impedance measurements with functional auditory outcomes and imaging-based evaluation of intracochlear tissue changes may provide a more comprehensive understanding of the clinical relevance of impedance modulation.

## 5. Conclusions

In conclusion, the present study demonstrates differences in postoperative electrode impedance dynamics associated with the addition of a preoperative postauricular GC injection to standard intraoperative intratympanic steroid administration. These findings are limited to electrophysiological observations of the electrode–tissue interface.

## Figures and Tables

**Table 1:** Demographic information in children.

	Control Group, *n* = 24	Treatment Group, *n* = 20	Z/X^2^	*p*
Age at CI (y)	3.88 (2.87, 4.88)	5.45 (3.64, 7.26)	−1.383 ^a^	0.167
Sex (Male: Female)	9:15	12:8	2.214 ^b^	0.137
Ear side (left: right)	5:19	8:12	1.925 ^b^	0.165
Duration of deafness (y)	3.88 (2.87, 4.88)	5.45 (3.64, 7.26)	−1.383 ^a^	0.167
* Presence of residual hearing (*n*, %)	1 (4.2%)	3 (15%)	0.516 ^c^	0.471
History of hearing aid use (*n*, %)	16 (66.7%)	10 (50%)	1.254 ^b^	0.263
Etiology of hearing loss (*n*, %)			/	
Idiopathic congenital hearing loss	19 (79.2%)	13 (65%)		0.503 ^d^
Cochlear malformation	3 (12.5%)	3 (15%)		
LVAS	2 (8.3%)	4 (20%)		

^a^ Mann–Whitney U test; ^b^ Chi-Squared test; ^c^ Continuity Correction Chi-square test; ^d^ Fisher’s exact probability test. * Residual hearing is defined as measurable low-frequency hearing thresholds detected by preoperative pure-tone audiometry.

**Table 2:** Demographic information in adults.

	Control Group, *n* = 15	Treatment Group, *n* = 10	Z/X^2^	*p*
Age at CI (y)	32.00 (29.00, 50.00)	30.00 (31.50, 48.25)	−0.083 ^a^	0.934
Sex (Male: Female)	6:9	6:4	/	0.428 ^b^
Ear side (left: right)	5:10	3:7	/	0.607 ^b^
Duration of deafness (y)	2.0 (5.0, 10.0)	3.5 (6.0, 15.0)	−0.363 ^a^	0.717
Presence of residual hearing (*n*, %)	7 (46.7%)	6 (60.0%)	/	0.688 ^b^
History of hearing aid use (*n*, %)	15 (100.0%)	10 (100.0%)	/	1.000 ^b^
Etiology of hearing loss (*n*, %)				
ISSHL	6 (40%)	4 (40%)		
Meningitis	1 (6.67%)	1 (10%)		
Otitis media	2 (13.33%)	3 (30%)	/	0.707 ^b^
Ototoxicity-related	1 (6.67%)	1 (10%)		
ILAHL	5 (33.33%)	1 (10%)		

^a^ Mann–Whitney U test; ^b^ Fisher’s exact probability test.

**Table 3:** Impedances across cochlear turns over time in children (kΩ).

Cohort	Time	Group	Regions		
			Apex	Middle	Base
Children	Intra-operative	Control	6.52 (4.57, 7.40)	5.20 (4.07, 6.10)	4.65 (3.79, 5.49)
		Treatment	5.98 (5.24, 6.98)	3.94 (3.34, 5.22)	3.95 (3.33, 5.11)
	1 Month	Control	8.50 ± 2.57	7.44 (6.15, 8.22)	6.77 (6.19, 7.65)
		Treatment	9.51 ± 1.50	6.77 (6.38, 8.61)	6.72 (5.46, 7.46)
	3 Months	Control	7.77 ± 2.02	6.38 ± 1.52	6.15 ± 1.60
		Treatment	8.29 ± 2.30	6.32 ± 2.37	5.59 ± 1.78
	6 Months	Control	7.55 ± 2.19	6.31 ± 1.49	6.07 ± 1.68
		Treatment	7.00 ± 1.89	5.26 ± 1.71	4.94 ± 1.75

**Table 4:** Impedances across cochlear turns over time in adults (kΩ).

Cohort	Time	Group	Regions		
			Apex	Middle	Base
Adults	Intra-operative	Control	11.32 ± 2.68	11.57 (8.28, 12.72)	10.73 (9.14, 11.32)
		Treatment	10.52 ± 1.98	10.80 (7.79, 11.96)	8.63 (6.56, 9.78)
	1 Month	Control	12.64 (11.26, 16.10)	13.80 (11.76, 15.16)	13.33 ± 1.68
		Treatment	13.57 (12.15, 14.460	11.60 (10.58, 13.91)	12.55 ± 1.81
	3 Months	Control	9.72 ± 1.86	10.33 ± 1.95	10.90 ± 2.15
		Treatment	9.26 ± 4.12	9.14 ± 1.50	9.94 ± 2.14
	6 Months	Control	8.88 ± 1.94	9.90 ± 1.87	10.14 ± 1.38
		Treatment	7.88 ± 3.50	8.32 ± 1.76	8.49 ± 1.51

**Table 5:** Linear mixed-effects model results in children.

Effects	dfd	dfn	*F*	*p*
Baseline impedance	1	66.3	19.29	0.000
Group	1	41.3	0.21	0.650
Turn	2	319	105.36	0.000
Time	2	318	54.27	0.000
Group × Turn	2	318	2.68	0.070
Group × Time	2	318	8.34	0.000
Turn × Time	4	318	0.32	0.862
Group × Turn × Time	4	318	0.26	0.903

**Table 6:** Adjusted mean impedance values and intervention effects by time point in children (kΩ).

Time	Group	Adjusted Mean Values (95%CI)	Intervention Effects * (95%CI)	*p*
1 month	Control	7.41 (6.81, 8.00)	−0.70 (−1.59, 0.19)	0.120
	Treatment	8.10 (7.44, 8.75)		
3 months	Control	6.59 (5.99, 7.18)	−0.43 (−1.32, 0.46)	0.343
	Treatment	7.02 (6.37, 7.68)		
6 months	Control	6.44 (5.84, 7.03)	0.50 (−0.39, 1.40)	0.264
	Treatment	5.95 (5.29, 6.61)		

* Intervention effect = control-treatment; positive values indicate lower impedance in the treatment group.

**Table 7:** Linear mixed-effects model results in adults.

Effects	dfd	dfn	*F*	*p*
Baseline impedance	1	47.5	4.64	0.036
Group	1	23.4	1.01	0.324
Turn	2	185	1.26	0.286
Time	2	184	103.28	0.000
Group × Turn	2	182	3.07	0.049
Group × Time	2	181	0.63	0.533
Turn × Time	4	184	0.46	0.762
Group × Turn × Time	4	181	0.52	0.724

**Table 8:** Adjusted mean impedance values and intervention effects by time point in adults (kΩ).

Cochlear Turn	Group	Adjusted Mean Values (95%CI)	Intervention Effects * (95%CI)	*p*
Apex	Control	10.49 (9.71, 11.26)	−0.406 (−1.652, 0.840)	0.515
	Treatment	10.89 (9.94, 11.85)		
Middle	Control	11.14 (10.38, 11.91)	1.355 (0.115, 2.596)	0.033
	Treatment	9.79 (8.83, 10.74)		
Base	Control	11.30 (10.54, 12.07)	0.630 (−0.610, 1.871)	0.311
	Treatment	10.67 (9.72, 11.63)		

* Intervention effect = control-treatment; positive values indicate lower impedance in the treatment group.

## Data Availability

The data cannot be shared publicly because of participant privacy issues. Researchers can access the data by submitting a suitable proposal to the corresponding author S.Z.

## Abbreviations

The following abbreviations are used in this manuscript:

CI	Cochlear Implantation
GC	Glucocorticoid
kΩ	Kiloohm (unit of electrical impedance)
MRI	Magnetic Resonance Imaging
CT	Computed Tomography
IV	Intravenous
PO	Per os (Oral)
SD	Standard Deviation
F	F-statistic (from ANOVA/F-test)
df	Degrees of freedom
SAS	Statistical Analysis System (software)
MED-EL	A Cochlear implant manufacturer
LVAS	Large vestibular aqueduct syndrome
ILAHL	Idiopathic long-term acquired hearing loss
ISSHL	Idiopathic sudden sensorineural hearing loss

## Appendix A

**Table A1:** Sensitivity analysis results with different model specifications in the pediatric study.

	Model Specifications	Effects	*F*	*p*
Model 1	Without baseline adjustment	Group	0.03	0.864
		Turn	103.87	0.000
		Time	53.87	0.000
		Group × Turn	3.01	0.051
		Group × Time	8.54	0.000
		Turn × Time	0.34	0.849
		Group × Turn × Time	0.34	0.854
Model 2	Analysis of change from baseline	Group	1.14	0.292
		Turn	101.50	0.000
		Time	51.88	0.000
		Group × Turn	1.93	0.147
		Group × Time	7.54	0.001
		Turn × Time	0.30	0.877
		Group × Turn × Time	0.19	0.944
Model 3	Including baseline interaction terms	Baseline impedance	16.89	0.000
		Group	0.12	0.729
		Turn	40.48	0.000
		Time	57.82	0.000
		Baseline impedance × Group	0.02	0.883
		Baseline impedance × Turn	5.43	0.005
		Group × Turn	1.54	0.216
		Group × Time	9.23	0.000
Model 4	Modeling time as a continuous predictor	Baseline impedance	19.65	0.000
		Group	4.90	0.031
		Turn	35.43	0.000
		Time	102.26	0.000
		Time × Group	16.57	0.000
		Time × Turn	0.48	0.618
		Group × Turn	2.70	0.068

**Table A2:** Sensitivity analysis results with different model specifications in the adult study.

	Model Specifications	Effects	*F*	*p*
Model 1	Without baseline adjustment	Group	2.45	0.131
		Turn	1.25	0.289
		Group × Turn	3.18	0.044
		Time	110.47	0.000
		Group × Time	0.61	0.545
		Turn × Time	0.83	0.509
		Group × Turn × Time	0.44	0.779
Model 2	Analysis of change from baseline	Group	0.27	0.608
		Turn	2.16	0.118
		Group × Turn	2.37	0.097
		Time	82.34	0.000
		Group × Time	0.67	0.512
		Turn × Time	0.38	0.824
		Group × Turn × Time	0.75	0.560
Model 3	Including baseline interaction terms	Baseline impedance	3.68	0.061
		Group	0.01	0.910
		Baseline impedance × Group	0.02	0.875
		Turn	5.53	0.005
		Baseline impedance × Turn	6.99	0.001
		Group × Turn	5.00	0.008
		Time	105.30	0.000
		Group × Time	0.67	0.513
		Turn × Time	0.38	0.821
		Group × Turn × Time	0.51	0.727
Model 4	Modeling time as a continuous predictor	Baseline impedance	6.76	0.013
		Group	0.21	0.648
		Turn	0.76	0.467
		Time	152.52	0.000
		Time × Group	0.20	0.658
		Time × Turn	0.51	0.601
		Group × Turn	0.18	0.840
		Time × Group × Turn	0.54	0.586
